# Toward nanoscopic cellular imaging by X-ray

**DOI:** 10.1093/nsr/nwaa206

**Published:** 2020-09-08

**Authors:** Jie Gao, Zhen Gu

**Affiliations:** Department of Bioengineering, University of California, Los Angeles, USA; State Key Laboratory of Medicinal Chemical Biology and College of Life Sciences, Nankai University, China; Department of Bioengineering, University of California, Los Angeles, USA; Jonsson Comprehensive Cancer Center and California NanoSystems Institute, University of California, Los Angeles, USA

X-ray spectroscopy has nanometer spatial resolution, long penetration depth and excellent elemental specificity. The power of synchrotron-based X-ray microscopy (XRM) has been demonstrated in assessing the structure and function of cells at the subcellular level [1]. However, currently available contrast agents or probes for XRM, such as metal or semiconductor nanoparticles, generally lack specificity and biocompatibility and thus limit its utility [2]. Newly developed genetic tags for electron microscopy (EM) provide EM contrast in cellular compartments *in situ* and enable intracellular specific protein imaging and spatially resolved proteomic mapping [3,4], but use of EM for whole cell imaging remains challenging because of low penetration capability.

**Figure 1.**
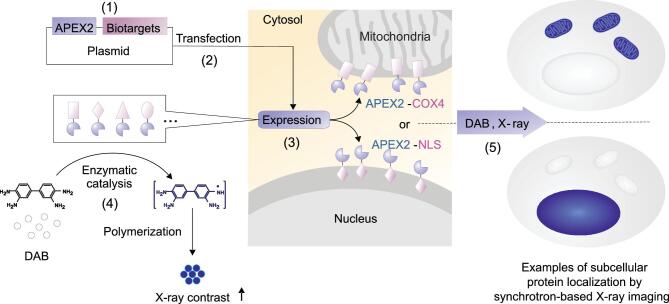
Schematic of genetically encoded tagging and X-ray cellular imaging for protein localization. Protein localization with XRM is achieved by (1) constructing fusion expression plasmids including APEX2 and biotargets, (2) transfecting cells with plasmids, (3) specific expressing of fusion proteins containing APEX2 and biotargets, (4) catalyzing the polymerization of DAB *in situ* into localized X-ray visible dense DAB polymers and (5) XRM imaging.

In a research article recently published in *NSR* [5], Fan, Zhu and coworkers reported a genetically encoded tagging system that could generate XRM reporters intracellularly, which were used for imaging organelles and specific proteins in cells at an ultra-high resolution of about 30 nm (Fig. 1). They firstly confirmed that an engineered ascorbate peroxidase (APEX2) could provide contrast enhancement for XRM by catalyzing local polymerization of diaminobenzidine (DAB). Polymerized DAB showed much higher contrast to the water background compared to DAB monomers, as shown in images obtained by synchrotron-based scanning transmission X-ray microscopy (STXM). To substantiate the utility of the APEX2 system for XRM imaging in mammalian cells, they constructed plasmids expressing APEX2 fused to different proteins. The proteins fused with APEX2 were either components of subcellular structures, such as nexin-43, *α*-tubulin and *β*-actin, or markers of organelles, including nuclear localization sequence (NLS) and galactosyltransferase. After staining with DAB monomers, the STXM images of transfected cells showed distinctly high-contrast structures with typical shapes of corresponding subcellular structures, namely gap junctions, microtubules, microfilaments, cell nucleus and Golgi apparatus, compared to cells without tagging. Of note, the X-ray tag showed enhanced photostability (almost no photo-bleaching after 10 frames of scans) compared to endogenous fluorescent tags (up to 28% decrease), which allowed repetitive scanning at high density and thus enabled imaging with high spatial and energy resolution. Furthermore, the XRM cell imaging based on this tagging system had approximately an order of magnitude improved spatial resolution (about 30 nm) when compared to the resolution of classic optical microscopy (about 200 nm).

The authors demonstrated two important applications of this genetic tagging and X-ray imaging system: it can be used to image subtle changes and refined structures of intercellular connections, which remains challenging for optical microscopy and electron microscopy because of their resolution limits; and it can achieve multi-color X-ray imaging by introducing different peroxidase tags and DAB substrates containing elements with distinguishable adsorption energies.

The work by the Fan and Zhu group provides a highly versatile platform technique for imaging targeted molecules and structures in cells with high specificity and resolution. Importantly, as part of the `Synchrotron for Neuroscience—an Asia Pacific Strategic Enterprise' (SYNAPSE) project, aiming to comprehensively map the neuronal network of the entire human brain, this genetically encoded X-ray tagging system can be adapted readily to visualize the neuron network at a subcellular level resolution.

***Conflict of interest statement*.** None declared.
